# Overexpression of Cucumber *Phospholipase D alpha* Gene (*CsPLDα*) in Tobacco Enhanced Salinity Stress Tolerance by Regulating Na^+^–K^+^ Balance and Lipid Peroxidation

**DOI:** 10.3389/fpls.2017.00499

**Published:** 2017-04-07

**Authors:** Tuo Ji, Shuzhen Li, Meili Huang, Qinghua Di, Xiufeng Wang, Min Wei, Qinghua Shi, Yan Li, Biao Gong, Fengjuan Yang

**Affiliations:** ^1^State Key Laboratory of Crop Biology, College of Horticulture Science and Engineering, Shandong Agricultural UniversityTai’an, China; ^2^Key Laboratory of Biology and Genetic Improvement of Horticultural Crops (Huanghuai Region), Ministry of AgricultureTai’an, China

**Keywords:** *CsPLD*α, transgenic tobacco, salt stress, ion homeostasis, lipid peroxidation

## Abstract

Plant phospholipase D (PLD), which can hydrolyze membrane phospholipids to produce phosphatidic acid (PA), a secondary signaling molecule, has been proposed to function in diverse plant stress responses. In this research, we characterized the roles of the cucumber *phospholipase D alpha* gene (*PLD*α, GenBank accession number EF363796) in growth and tolerance to short- and long-term salt stress in transgenic tobacco (*Nicotiana tabacum*). Fresh and dry weights of roots, PLD activity and content, mitogen activated protein kinase (MAPK) gene expression, Na^+^–K^+^ homeostasis, expression of genes encoding ion exchange, reactive oxygen species (ROS) metabolism and osmotic adjustment substances were investigated in wild type (WT) and *CsPLD*α-overexpression tobacco lines grown under short- and long-term high salt (250 mM) stress. Under short-term stress (5 h), in both overexpression lines, the PA content, and the expression levels of MAPK and several genes related to ion exchange (*NtNHX1*, *NtNKT1*, *NtHAK1*, *NtNHA1*, *NtVAG1*), were promoted by high PLD activity. Meanwhile, the Na^+^/K^+^ ratio decreased. Under long-term stress (16 days), ROS scavenging systems (superoxide dismutase, peroxidase, catalase, ascorbate peroxidase activities) in leaves of transgenic lines were more active than those in WT plants. Meanwhile, the contents of proline, soluble sugar, and soluble protein significantly increased. In contrast, the contents of O_2_^•−^ and H_2_O_2_, the electrolytic leakage and the accumulation of malondialdehyde in leaves significantly decreased. The root fresh and dry weights of the overexpression lines increased significantly. Na^+^–K^+^ homeostasis had the same trend as under the short-term treatment. These findings suggested that *CsPLD*α-produced PA can activate the downstream signals’ adaptive response to alleviate the damage of salt stress, and the main strategies for adaptation to salt stress are the accumulation of osmoprotective compounds, maintaining Na^+^–K^+^ homeostasis and the scavenging of ROS, which function in the osmotic balancing and structural stabilization of membranes.

## Introduction

Soil salinization is a severe problem facing agricultural production, affecting at least 20% of the irrigated land that has become more serious in recent years (Yamaguchi and Blumwald, 2005; Munns and Tester, 2008). The soil in China is severely affected by this problem, and, because of the extent of the affected area, salinization poses a serious threat to regional agricultural development (Li et al., 2014). High salinity in soil causes osmotic stress, ionic toxicity, and ionic imbalances; therefore, it seriously affects plant growth and development (Kim et al., 2007; Munns and Tester, 2008). However, plants have developed survival strategies to protect themselves from detrimental surroundings. Many signaling pathways are activated when plants are subjected to salt stress, including ion transporters and channels, such as the Na^+^/H^+^ transporter, and Ca^2+^ and K^+^ inward and outward channels, protein kinases and lipid-signaling, as well as the expression of salt stress-responsive genes, such as *salt overly sensitive (SOS)*, *NHX1*, and *LEAs* (Hasegawa et al., 2000; Kazi et al., 2013). In *Arabidopsis*, proton pumps generate an electrochemical gradient that is subsequently utilized by the tonoplast Na^+^/H^+^ antiporter *AtNHX1* to sequester Na^+^ in vacuoles to enhance its salt tolerance (Shiratake and Martinoia, 2007). The transcript levels of tomato *VHA-A1* and *-A2* are elevated by salt stress (Bageshwar et al., 2005).

Phospholipase D is a phosphatidyl choline-hydrolyzing enzyme that generates PA, a lipid second messenger, which can activate the downstream signals’ adaptive responses to alleviate the damage of stress (Wang, 2005; Bargmann and Munnik, 2006; Jang et al., 2012; Kolesnikov et al., 2012; Ruelland et al., 2015; Hong et al., 2016; Huo et al., 2016). PLDs are involved in high salinity stress. Cytoskeletal reconstruction assists plant resistance to salt stress from the environment, and PLDs may play a main role in cytoskeletal-mediated stress signal perception and transduction, which has a great influence in cytoskeletal reorganization (Gardiner et al., 2001). In tomato cell suspension cultures, tomato (*Lycopersicon esculentum*) α-class PLD *(LePLDα1)* gene expression is induced under NaCl stress (Bargmann et al., 2009). In *Arabidopsis*, both *AtPLD*δ and *AtPLDα1* gene expression levels increase under high salinity stress. Additionally, *AtPLDα1* and *AtPLD*δ single and double knock-out mutants exhibit enhanced sensitivity to high salinity stress, and their combined deletion renders plants more sensitive than the single mutants alone, displaying strongly reduced growth (Laxalt et al., 2001). The increased expression of *PLDε* also enhances root growth and biomass accumulation under severe nitrogen deprivation and salt stress in *Arabidopsis* (Hong et al., 2009). The increased expression of *AtPLDα1* in guard cells of *Brassica napus* decreased the water loss and improved biomass accumulation under hyperosmotic stress conditions, including drought and high salinity (Lu et al., 2013). In addition, *Arabidopsis PLDa3* knock-out mutants display hypersensitivity to salt stress, while the overexpression plants exhibit more resistant to this stress (Hong et al., 2008). The heterologous expression of the phospholipase Dα gene from *Ammopiptanthus nanus* (*AnPLD*α) improved salt tolerance of an *Arabidopsis plda1* knock-out mutant and positively regulated the expression of the *AtABI*, *AtNCED*, *AtRD29A*, *AtRD29B*, and *AtADH* genes (Yu et al., 2015). Thus, PLDs may play important roles in plant salt tolerance.

In past research, we showed that the expression of a cucumber *phospholipase D alpha* gene (*CsPLD*α) was induced by salt and drought stresses in the roots and leaves. To further study the roles of *CsPLD*α in regulating plant tolerance to hyperosmotic stress, transgenic tobacco plants constitutively overexpressing *CsPLD*α were produced. And the overexpression of *CsPLD*α significantly enhanced the seed germination rate and plant growth under high salinity, polyethylene glycol and abscisic acid treatments. Here, we further investigated the expression pattern of *CsPLD*α under salinity stress. *CsPLD*α-produced PA can activate the downstream signals’ adaptive response to alleviate the damage of salt stress, and the main strategies for adaptation to salt stress are the accumulation of osmoprotective compounds, maintaining Na^+^–K^+^ homeostasis and the scavenging of ROS. The results provide information for further exploration of the functions of *CsPLD*α in the salinity-resistance pathway of plants.

## Materials and Methods

### Plant Materials, Growth Condition, and NaCl-Stress Treatment

Tobacco (*Nicotiana tabacum* cv. NC89) was used for salt stress response assays, and the transgenic lines (resulting from self-fertilization), ‘T_1_-68’ and ‘T_1_-71,’ were produced during previous research (Li et al., 2015). Based on the germination results, the seeds of WT tobacco plants were sown 2 days earlier than those of transgenic plants. All of the seeds were sterilized in 2.5% NaClO and were sown into plastic plots (10 cm × 10 cm). Then, 2 weeks later, robust seedlings were transferred to plastic plugs (50 holes) filled with nursery substrate (peat:vermiculite:perlite = 2:1:1). The above experiment was carried out in a controlled environment in an illuminated incubator with an air temperature of 28°C during the day and 23°C during the night, a light intensity of 140 mmol m^-2^ s^-1^, and a relative humidity of 60%. Four weeks later, batches of five seedlings of WT and *CsPLD*α overexpression plants (‘T_1_-68’ and ‘T_1_-71’) were transferred to a 5-L (33 cm × 25 cm × 11 cm) plastic tank filled with an aerated complete nutrient solution (pH 6.0–6.5) containing 4.0 mM Ca(NO_3_)_2_⋅4H_2_O, 6 mM KNO_3_, 1 mM NH_4_H_2_PO_4_, 2 mM MgSO_4_⋅7H_2_O, 46.3 μM H_3_BO_3_, 10 μM MnSO_4_⋅H_2_O, 0.77 μM ZnSO_4_⋅7H_2_O, 0.02 μM (NH_4_)_6_Mo_7_O_2_⋅4H_2_O, 0.32 μM CuSO_4_⋅5H_2_O, and 54 μM EDTA-Fe Na in a glass greenhouse at Shandong Agricultural University. The experiment was carried out under natural conditions with an air temperature of 25–30°C during the day and 18–25°C at night. After 2 weeks, young tobacco seedlings were used as experimental materials for the treatments as described below.

### Short-Term NaCl-Stress Treatment

According to the method described by Gaxiola et al. (2001), the nutrient solution was supplemented with NaCl, incrementally increasing with each successive watering from 50, through 100, 150 and 200, to a final concentration of 250 mM NaCl. Tanks were arranged in a completely randomized block design with three replicates, providing a total of three tanks with a total of 15 plants per treatment. The samples were collected after 0 and 5 h treatments. The PLD activity and PA content in leaves, the expression of endogenous *NtPLDα1* and mitogen activated protein kinase (*NtMAPK*), the Na^+^ and K^+^ contents and the expression of several genes related to ion exchange (*NtNHX1*, *NtNKT1*, *NtHAK1*, *NtNHA1*, and *NtVAG1*) in leaves and roots were measured.

### Long-Term NaCl-Stress Treatment

Based on the aforementioned method, when NaCl reached the treatment concentration, replacing the nutrient solution to form a final NaCl concentration of 250 mM, the nutrient solutions in all the plastic tanks were completely renewed every 3 days. Tanks were arranged in a completely randomized block design with five replicates, providing a total of five tanks with a total of 25 plants per treatment. The samples were collected before treatment (CK) and after 8 and 16 days of the 250 mM NaCl treatment. Electrolyte leakage (EL), malondialdehyde (MDA) content, antioxidant enzyme [superoxide dismutase (SOD), peroxidase (POD), ascorbate peroxidase (APX), and catalase (CAT)] activities, ROS (O_2_^•−^ and H_2_O_2_) generation, Na^+^ and K^+^ contents, and the contents of proline, soluble protein, and soluble sugar in leaves were measured. In addition, fresh weights (FWs) and dry weights (DWs) of roots under conditions of control and stress (250 mM NaCl) were measured 16 days after treatment.

### PLD Activity Analysis and PA Content Determination

Cytoplasmic extracts from treated plant leaves were measured for PLD activity using a PLD ELISA kit as per the manufacturer’s instructions (Bangyi, China). The PA content was analyzed using a PA ELISA kit per the manufacturer’s instructions (Fangcheng, China).

### Measurement of Na^+^ and K^+^ Contents

The Na^+^ and K^+^ contents of leaves and roots were determined using a flame photometer (F-300, Shanghai Metash Instruments, Co. Ltd, Shanghai, China) (Essah et al., 2003).

### Quantitative Real-Time PCR

Total RNA was extracted from young leaves and roots of tobacco using the TRIzol method according to the manufacturer’s instructions (CWBIO). Total RNA (1 μg) from each sample was used as a template for the reverse transcription reaction to synthesize cDNA using the TransScript One-Step gDNA Removal and cDNA Synthesis SuperMix (TransGen). The *NtActin* primers, 5′-TTAAAGAGAAACTGGCATATGTTG-3′ (forward) and 5′-GCCCATCTGGTAACTCATAGC-3′ (reverse), were used to amplify the internal control. The PCR primers were designed to avoid the conserved region and to amplify products of 150- to 300-bp products. Primer sequences are shown in detail in Supplementary Table S1. Quantitative RT-PCR was performed using the TransStart^®^ Tip Green qPCR SuperMix, and 25 μL of the reaction system was mixed for the determination of salt stress-associated gene expression using a ABI 7500 RT PCR instrument (Thermo Fisher Scientific, USA) as described in the literature (Li et al., 2012). The procedure of Real-time PCR was: 95°C for 10 min, followed by 40 cycles of 95°C for 15 s, 60°C for 1 min. Each expression profile was independently verified in three replicate experiments. Data were analyzed using SDS 2.0 software (ABI), and the relative gene expression levels were calculated using the method of 2^-ΔΔCt^ (Livak and Schmittgen, 2001).

### Determination of Root FW and DW

Under conditions of control and stress (250 mM NaCl) after 16 days of treatment, three plants each of WT, ‘T_1_-68’ and ‘T_1_-71’ were collected. The FWs of tobacco roots were measured immediately. Then, the root samples were dried at 80°C for 48 h and then measured to obtain the DWs.

### ROS Accumulation, EL and Lipid Peroxidation Analysis

The histochemical staining of O_2_^•−^ and H_2_O_2_ was performed as previously described (Xia et al., 2009). The O_2_^•−^ productivity rate and H_2_O_2_ content were quantified according to Patterson (Rauckman et al., 1979; Patterson et al., 1984). The EL and MDA content were determined according to Wang et al. (2010).

### Determination of Antioxidant Enzyme Activities

Superoxide dismutase activity was assayed by measuring its ability to inhibit the photochemical reduction of nitrobluetetrazolium following the method of Stewart and Bewley (1980). CAT activity was measured as the decline in absorbance at 240 nm due to the decrease in the extinction of H_2_O_2_, according to the method of Patra et al. (1978). POD activity was measured by the increase in absorbance at 470 nm due to guaiacol oxidation (Nickel and Cunningham, 1969). APX activity was measured by the decrease in absorbance at 290 nm as ascorbic acid was oxidized according to previous studies (Nakano and Asada, 1981).

### Determination of Proline, Soluble Sugar, and Soluble Protein Contents

Fresh leaf samples of 0.5 g were extracted with 5 mL of 3% sulfo-salicylic acid at 100°C for 10 min with shaking. The other processes were performed as previously described (Xu et al., 2011). Soluble sugar was measured by the sulfuric acid–anthrone colorimetry method (Dubois et al., 1951). The protein content was measured using bovine serum albumin as the standard, according to Bradford (1976).

### Statistical Analyses

Values presented are means ±1 standard deviation (SD) of three replicates. Statistical analyses were carried out by analysis of variance (ANOVA) using SAS (SAS Institute, Cary, NC, USA) software. Differences between treatments were separated by the least significant difference (LSD) test at *P* < 0.05 and *P* < 0.01.

## Results

### PLD Activity, PA Content, and *NtPLDα1* Expression under Short-Term NaCl Stress

**FIGURE 1:**
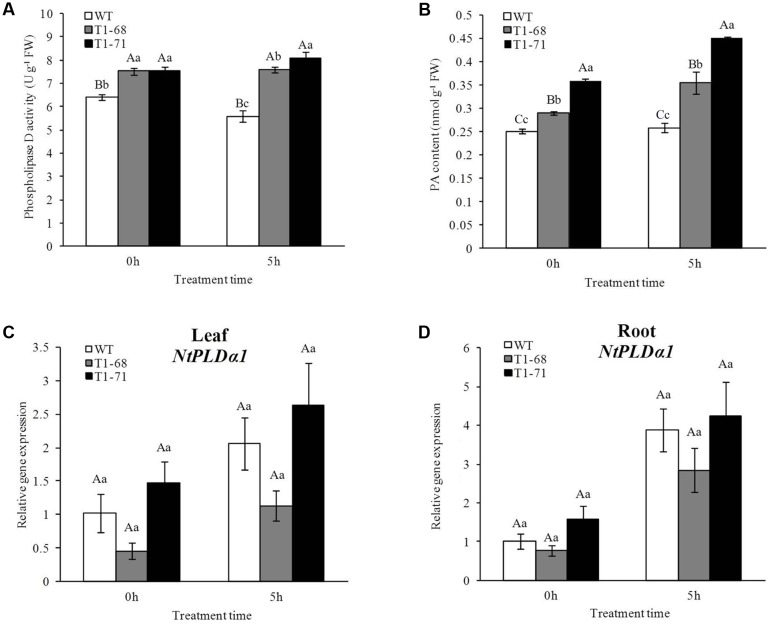
**Effects of short-term NaCl stress on phospholipase D activity** **(A)**, PA content **(B)** in leaves, and the expression levels of *NtPLDα1* in leaves **(C)** and roots **(D)** of both WT and transgenic tobacco. Values are means ± SD (*n* = 3). Mean values followed by different capital letters (A–C) and small letters (a–c) are significantly different (*P* < 0.01) and (*P* < 0.05), respectively.

As shown in **Figure 1A**, PLD activity in transgenic plant leaves increased as the 250 mM NaCl treatment time increased, while there was a decrease in the WT plants. When the NaCl concentration reached 250 mM (0 h) gradually, PLD activities in transgenic plants of ‘T_1_-68’ and ‘T_1_-71’ leaves were both 17.9% significantly higher than that of WT plants. After 5 h of the 250 mM NaCl treatment, the PLD activities in ‘T_1_-68’ and ‘T_1_-71’ leaves were 36.3 and 44.9% higher, respectively, than of the activity in WT plants. Moreover, to determine the involvement of endogenous *NtPLDα1* in salt responses, the expression of *NtPLDα1* in leaves and roots of WT and transgenic plants was also measured (**Figures 1C,D**). The results indicated that there was no difference between WT and transgenic plants under short-term NaCl stress. Thus, the overexpression of *CsPLD*α could enhance the sensitivity of plants to high salinity stress in a short time.

The change in the PA content was similar to that of the PLD activity (**Figure 1B**). The 250 mM NaCl treatment for 5 h significantly induced PA formation in *CsPLD*α-overexpression plants compared with WT plants. The PA content in ‘T_1_-68’ and ‘T_1_-71’ transgenic plants increased by 37.7 and 74.3%, respectively. When the NaCl concentration reached 250 mM (0 h) gradually, the PA content in leaves of ‘T_1_-68’ and ‘T_1_-71’ transgenic plants was also 15.8 and 43.4% higher, respectively, than its content in WT plants. As a result, accumulated PA could generate a range of reactions, especially triggering the salt-tolerance pathway in tobacco plants.

### Expression of *NtMAPK* under Short-Term NaCl Stress

**FIGURE 2:**
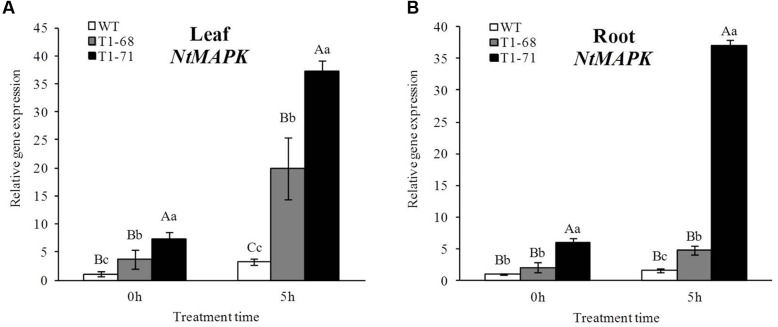
**Effects of short-term NaCl stress on the relative expression of *NtMAPK* in leaves** **(A)** and roots **(B)** of both WT and transgenic tobacco. Values are means ± SD (*n* = 3). Mean values followed by different capital letters (A–C) and small letters (a–c) are significantly different (*P* < 0.01) and (*P* < 0.05), respectively.

The MAPK cascades are major pathways by which extracellular signals, such as growth factors, hormones and stress stimuli, are transduced into intracellular responses in plants. To identify the relationship between the *CsPLD*α and *MAPK*, the expression of *NtMAPK* in leaves and roots of WT and transgenic plants were detected. **Figure 2** shows that the mRNA abundances of the *NtMAPK* gene in both leaves and roots of overexpression plants were remarkable induced after the 5 h treatment. Compared with those of WT plants, the levels of expression in the leaves and roots of ‘T_1_-68’ and ‘T_1_-71’ were higher by 2.5 or 3.0 times (**Figure 2A**) and 1.7 or 4.8 times (**Figure 2B**), respectively. When the NaCl concentration reached 250 mM (0 h) instantly, the transcript levels of MAPK gene in leaves and roots of transgenic plants did not undergo relatively large increases compared with the 5 h treatment, however, there was significant difference between transgenic and WT plants. Thus, in general, *NtMAPK* may be an important indicator in tobacco salt tolerance.

### Expression of Genes Related to Ion Exchange under Short-Term NaCl Stress

**FIGURE 3:**
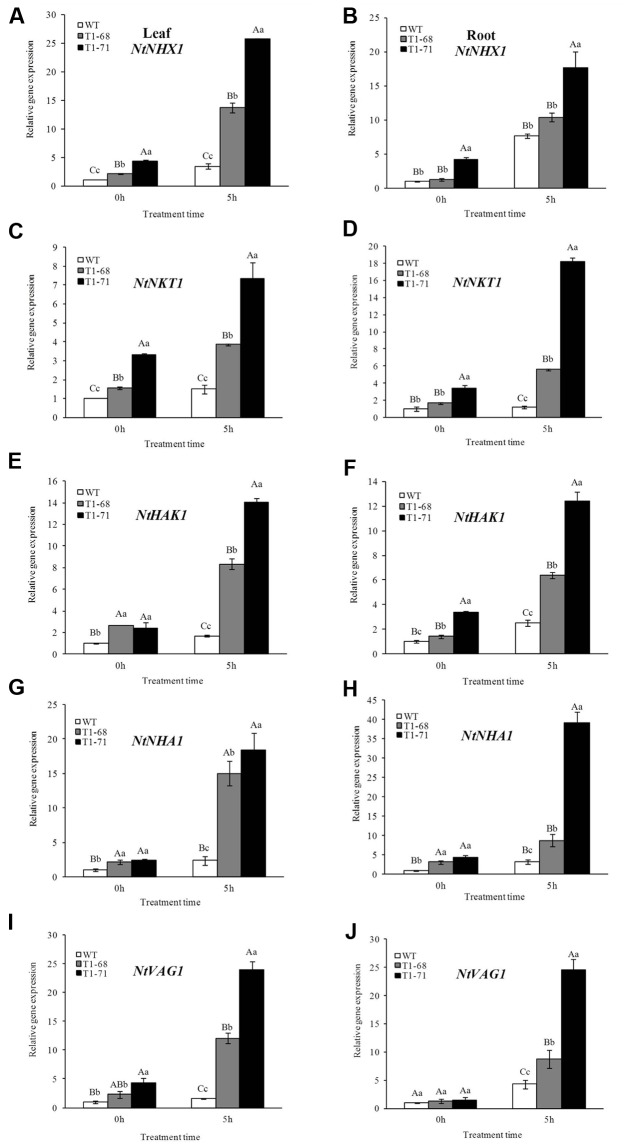
**Effects of short-term NaCl stress on the expression levels of ion exchange-related genes, including *NtNHX1*, *NtNKT1*, *NtHAK1*, *NtNHA1* and *NtVAG1*, in leaves** **(A,C,E,G,I)** and roots **(B,D,F,H,J)** of WT and transgenic tobacco. Values are means ± SD (*n* = 3). Mean values followed by different capital letters (A–C) and small letters (a–c) are significantly different (*P* < 0.01) and (*P* < 0.05), respectively.

To determine the involvement of *CsPLD*α in salt responses to modulate Na^+^–K^+^ homeostasis, RT-PCR was conducted to analyze the expression levels of ion exchange-related genes, including *NtNHX1*, *NtNKT1*, *NtHAK1*, *NtNHA1* and *NtVAG1*, in leaves and roots of transgenic and WT plants. *NtNHX1*, a major gene controlling sodium ion regionalization, could prompt the transportation of Na^+^ into vacuoles. *NtNKT1* is a gene encoding a K^+^ channel protein, while *NtHAK1* encodes a K^+^ transporter protein. Both *NtNHA1* and *NtVAG1* belong to proton pump genes, with one being located on the cytoplasmic membrane and the other on the vacuolar membrane. As shown in **Figures 3A–J**, after 5 h of stimulation with stress, the mRNA abundances of these regulatory genes in both roots and leaves of transgenic plants were dramatically induced compared with that of WT plants. When the NaCl concentration reached 250 mM (0 h) gradually, the transcript levels of genes related to ion exchange in leaves and roots of transgenic plants did not undergo a relatively large rise compared with after the 5 h of treatment.

### Homeostasis of Na^+^ and K^+^ under Short-Term NaCl Stress

**FIGURE 4:**
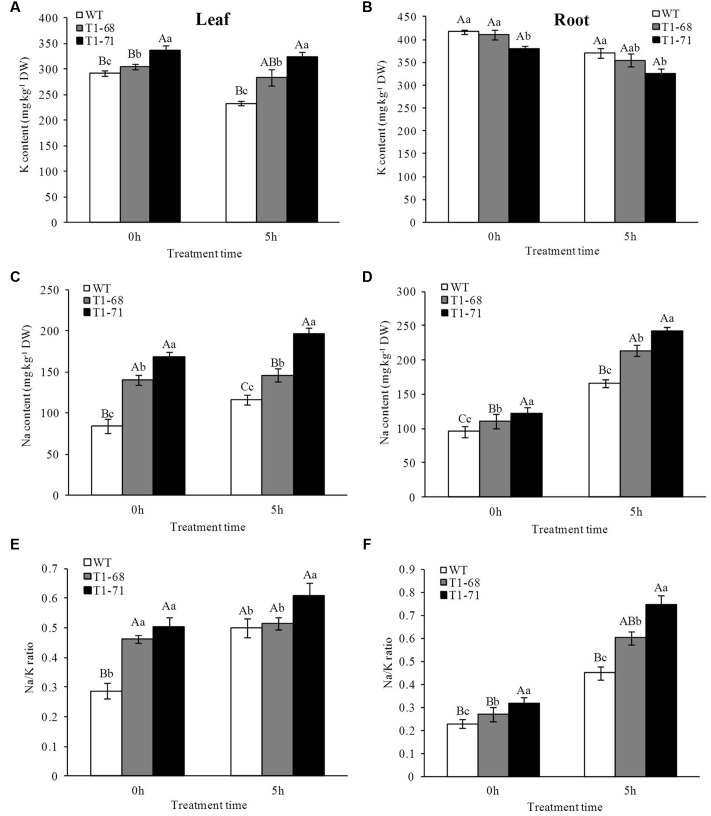
**Effects of short-term NaCl stress on K content** **(A,B)**, Na content **(C,D)** and Na/K ratio **(E,F)** in both WT and transgenic tobacco leaves **(A,C,E)** and roots **(B,D,F)**. Values are means ± SD (*n* = 3). Mean values followed by different capital letters (A–C) and small letters (a–c) are significantly different (*P* < 0.01) and (*P* < 0.05), respectively.

The significant changes in the expression of ion exchange-related genes resulted in alterations in the Na^+^ and K^+^ contents. Treatment with 250 mM NaCl for 5 h caused an obvious decrease in K^+^ content in roots compared with treatment for 0 h (**Figure 4B**). This decrease in ‘T_1_-68’ (13.6%) and ‘T_1_-71’ (14.5%) transgenic plants was more significant than that in WT plants (11.0%). Meanwhile, the K^+^ content in the roots of ‘T_1_-68’ and ‘T_1_-71’ transgenic plants was lower by 4.3 and 12.4%, respectively, than that of WT. Additionally, the K^+^ content in leaves was also decreased after treatment (**Figure 4A**). The decrease in the WT plants (20.0%) was more significant than that in the ‘T_1_-68’ (7.1%) and ‘T_1_-71’ (3.7%) transgenic plants. However, the K^+^ content in the leaves of ‘T_1_-68’ and ‘T_1_-71’ transgenic plants was 21.4 and 38.6% higher, respectively, than that of the WT.

Distinct from the altered patterns in the K^+^ content, the Na^+^ content was increased in both leaves and roots after treatment (**Figures 4C,D**). The transgenic tobacco cells absorbed more Na^+^ than WT cells after 0 and 5 h of the 250 mM NaCl treatment. Additionally, the Na^+^ contents in leaves and roots of ‘T_1_-68’ and ‘T_1_-71’ transgenic plants were significantly higher by 25.0 or 68.0% and 28.0 or 45.5%, respectively, after 5 h of the 250 mM NaCl treatment.

Based on the above findings, the ratio of Na^+^/K^+^ was calculated, and the results revealed that the ratio increased in both leaves and roots as the treatment time increased, and the ratio of the Na^+^/K^+^ concentration in transgenic plants was higher than in WT plants, especially in roots after 5 h of the 250 mM NaCl treatment (**Figures 4E,F**). The ratios of Na^+^/K^+^ in the roots of ‘T_1_-68’ and ‘T_1_-71’ transgenic plants were significantly higher by 33.7 and 66.1%, respectively, after 5 h of the 250 mM NaCl treatment. These findings suggested that *CsPLD*α-overexpression tobacco plants absorbed more Na^+^ than K^+^, which resulted in an increased ratio of Na^+^/K^+^ and consequently maintained Na^+^–K^+^ homeostasis. Combined with the results obtained from the expression analyses of the ion exchange genes, we concluded that *CsPLD*α negatively regulated salinity stress through the regulation of key genes involved in the maintenance of ion homeostasis at the transcriptional level and the resistance of salinity-induced osmotic stress over a short time.

### FW and DW under Long-Term NaCl Stress

**FIGURE 5:**
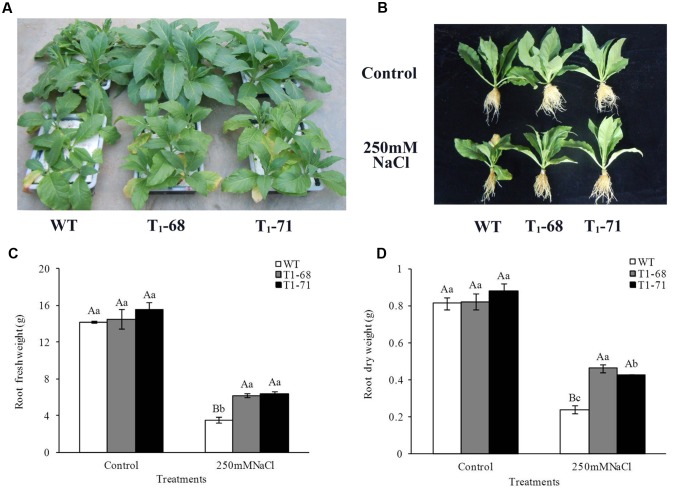
**Assessment of long-term NaCl stress in WT and transgenic tobacco seedlings.** Phenotypes of WT and transgenic seedlings under conditions of control and stress (250 mM NaCl). Photographs were taken after 16 days of treatment **(A,B)**. Root fresh weights **(C)** and dry weights **(D)** of WT and transgenic tobacco seedlings were measured after 16 days of stress (250 mM NaCl), separately. Values are means ± SD (*n* = 3). Mean values followed by different capital letters (A–C) and small letters (a–c) are significantly different (*P* < 0.01) and (*P* < 0.05), respectively.

After 16 days of treatment, the photographs showed that overexpression of *CsPLD*α in tobacco significantly promoted the growth of seedlings under 250 mM NaCl stress (**Figures 5A,B**). The root growth of both WT and transgenic seedlings were severely suppressed during the 16 days exposure to 250 mM NaCl. In particular, the growth of WT seedlings was more significantly inhibited. The FWs (**Figure 5C**) and DWs (**Figure 5D**) of WT, ‘T_1_-68’ and ‘T_1_-71’ roots were significantly lower by 301.3, 134.0 and 141.9%, and 239.2, 77.9, and 106.5%, respectively, compared with the control. The root FWs and DWs of ‘T_1_-68’ and ‘T_1_-71’ transgenic plants increased by 75.5 or 81.3% and 92.9 or 77.6%, respectively, compared with WT plants after undergoing the 16-days treatment. Thus, the transgenic tobacco plants exhibited stronger capabilities of resisting salt stress, while *CsPLD*α may play an important role in alleviating the stress damage caused by high salt.

### PLD Activity under Long-Term NaCl Stress

**FIGURE 6:**
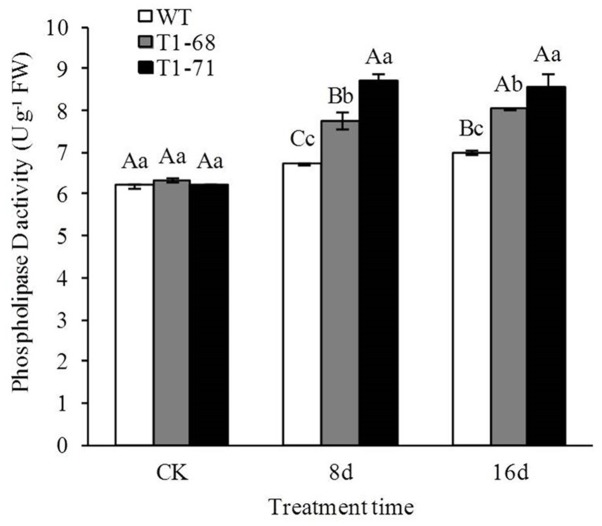
**Effects of long-term NaCl stress on phospholipase D activity in both WT and transgenic tobacco leaves.** Values are means ± SD (*n* = 3). Mean values followed by different capital letters (A–C) and small letters (a–c) are significantly different (*P* < 0.01) and (*P* < 0.05), respectively.

**Figure 6** shows that the PLD activity levels were not significantly different between WT and transgenic plants before treatment (CK). However, the PLD activities in transgenic plants were induced to a greater degree than that in the WT under high NaCl conditions. The PLD activities in leaves of ‘T_1_-68’ and ‘T_1_-71’ transgenic plants were significantly higher by 15.3 and 29.3%, respectively, than that in WT after 8 days of the 250 mM NaCl treatment. In addition, the high salt treatment slightly enhanced the PLD activities in WT and transgenic plants as the treatment time increased. Thus, PLD may be positively induced by salt stress and *CsPLD*α-overexpression may have sensitized plant responses to the long-term salt stress.

### Homeostasis of Na^+^ and K^+^ under Long-Term NaCl Stress

**FIGURE 7:**
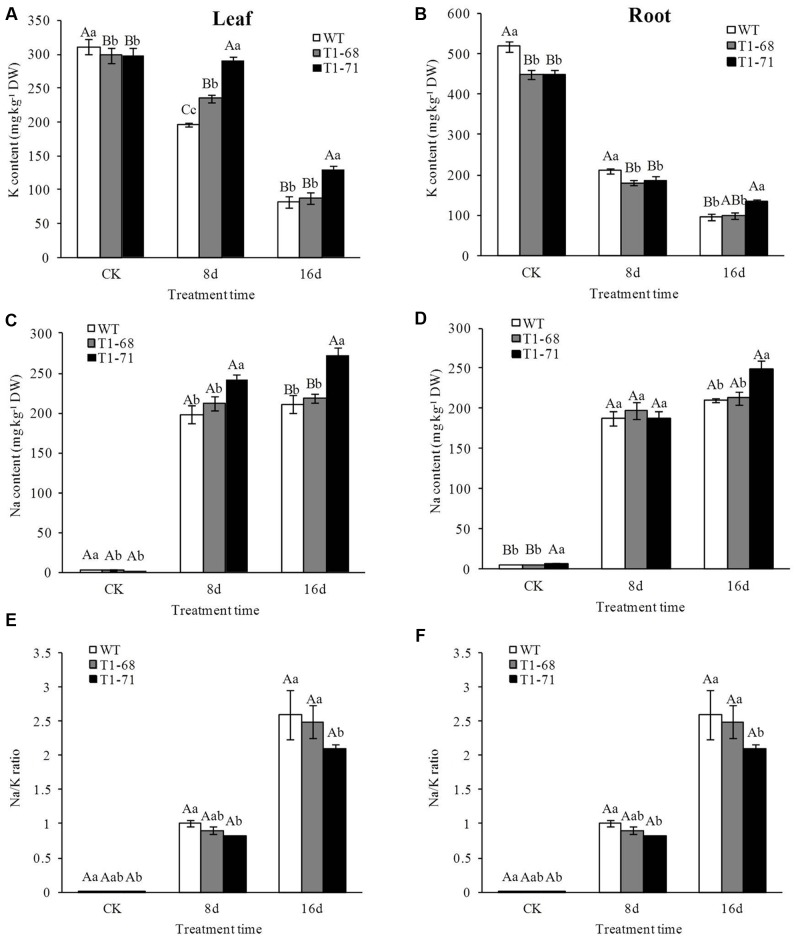
**Effects of long-term NaCl stress on K content** **(A,B)**, Na content **(C,D)** and Na/K ratio **(E,F)** in both WT and transgenic tobacco leaves **(A,C,E)** and roots **(B,D,F)**. Values are means ± SD (*n* = 3). Mean values followed by different capital letters (A–C) and small letters (a–c) are significantly different (*P* < 0.01) and (*P* < 0.05), respectively.

Under a long-time exposure to salts, Na^+^ concentrations in both the leaves and roots of WT and transgenic plants increased significantly (**Figures 7C,D**), while the K^+^ content decreased as shown in **Figures 7A,B**. Compared with WT plants, Na^+^ content after the 16-days treatment increased by more than 29.3% in leaves, but only 17.8% in roots, of ‘T_1_-71’ transgenic plants. Meanwhile, the K^+^ content increased by 57.7% in leaves and 40.6% in roots. Consequently, the Na^+^/K^+^ ratio increased in both the leaves and roots after 16 days of salt stress (**Figures 7E,F**). Unlike WT plants, both transgenic plants still maintained the highest levels of Na^+^ or K^+^ in their organisms, but the lowest Na^+^/K^+^ ratio after the 16-days salt stress, and the difference was more distinct in the ‘T_1_-71’ plants. Thus, the patterns of ion influx and efflux in all of the tobacco lines were consistent when the responses to salt stress. However, the overexpression of *CsPLD*α could protect against stress-induced changes in ion concentrations to maintain Na^+^–K^+^ homeostasis during a long-term treatment.

### EL and MDA under Long-Term NaCl Stress

**FIGURE 8:**
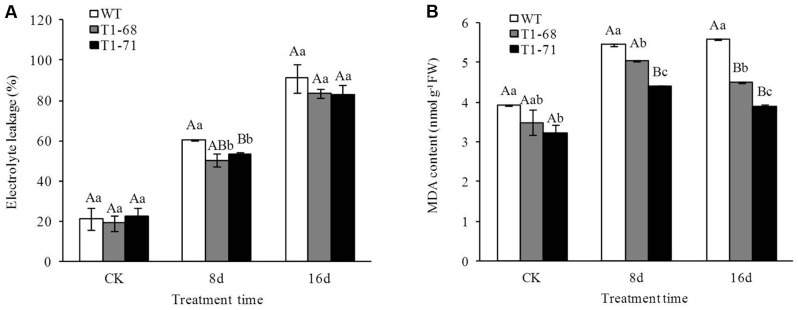
**Effects of long-term NaCl stress on electrolyte leakage (EL)** **(A)** and malondialdehyde (MDA) content **(B)** in both WT and transgenic tobacco leaves. Values are means ± SD (*n* = 3). Mean values followed by different capital letters (A–C) and small letters (a–c) are significantly different (*P* < 0.01) and (*P* < 0.05), respectively.

**Figure 8** shows that the 250 mM NaCl treatment generated more EL and MDA than the CK. Compared with WT lines, the EL of ‘T_1_-68’ in leaves decreased by 16.8% and that of ‘T_1_-71’ significantly decreased by 11.7% (*P* < 0.05) after 8 days of the 250 mM NaCl treatment (**Figure 8A**). The ELs in ‘T_1_-68’ and ‘T_1_-71’ transgenic plant leaves decreased by 8.4 and 8.9%, respectively, after the 16-days treatment; however, there were no significant differences between the transgenic and WT lines (*P* < 0.05).

After undergoing the same procedures, the MDA content in ‘T_1_-68’ and ‘T_1_-71’ transgenic plant leaves decreased by 19.3 and 30.0%, respectively, after 16 days of the 250 mM NaCl treatment (**Figure 8B**). This was a trend distinct from that of the EL. After 16 days of 250 mM NaCl, the MDA content in transgenic tobacco plants was lower than that 8 days after treatment. However, the MDA content in WT plants was maintained at a stable level. Thus, the expression of *CsPLD*α in tobacco plants enhanced the tolerability of plants to the salt stress associated with membrane lipid peroxidation.

### Overexpression of *CsPLD*α Alleviated Oxidative Stress Induced by Long-Term NaCl Stress and Increased Antioxidant System Functions in Tobacco Leaves

**FIGURE 9:**
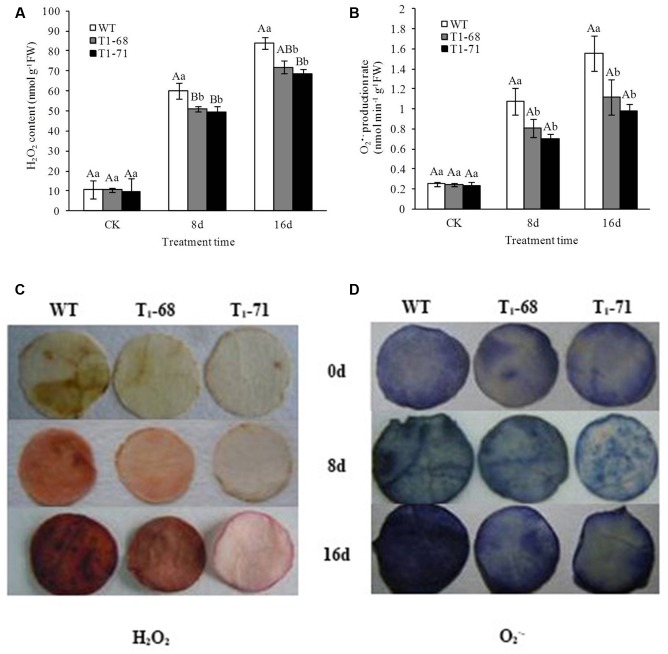
**Effects of long-term NaCl stress on H_2_O_2_ content** **(A)**, O_2_^•−^ production rate **(B)**, image of H_2_O_2_ content **(C)** and O_2_^•−^ production rate **(D)** in both WT and transgenic tobacco leaves. Values are means ± SD (*n* = 3). Mean values followed by different capital letters (A–C) and small letters (a–c) are significantly different (*P* < 0.01) and (*P* < 0.05), respectively.

With time, after NaCl stimulation, abundant levels of H_2_O_2_ and O_2_^•−^ accumulated in the plants and peaked at day 16 (**Figures 9A–D**). Overall, the degrees of H_2_O_2_ and O_2_^•−^ accumulation in the leaves of ‘T_1_-68’ and ‘T_1_-71’ plants were lower than in WT plants. The H_2_O_2_ contents in leaves of ‘T_1_-68’ and ‘T_1_-71’ lines decreased by 14.2 and 18.1%, respectively, when compared with WT plants. As for O_2_^•−^, the production rated in leaves of ‘T_1_-68’ and ‘T_1_-71’ plants decreased by 28.2 and 36.9%, respectively, compared with in leaves of WT plants.

**FIGURE 10:**
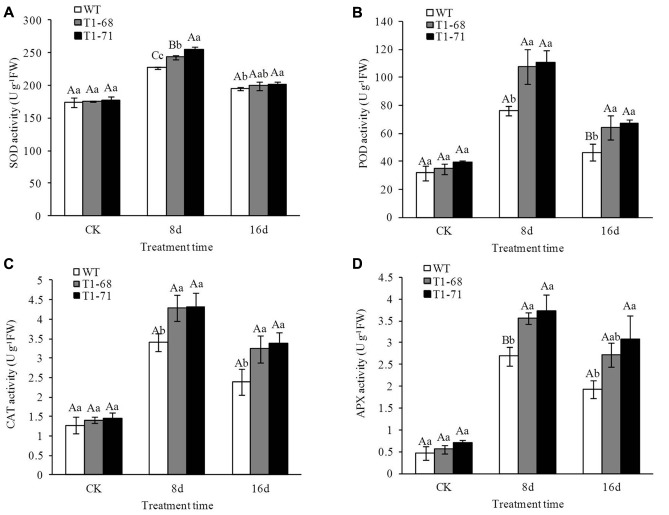
**Effects of long-term NaCl stress on the activities of SOD** **(A)**, POD **(B)**, CAT **(C)**, and APX **(D)** in both WT and transgenic tobacco leaves. Values are means ± SD (*n* = 3). Different capital letters indicate significant difference (*P* < 0.01), and different small letters indicate significant difference (*P* < 0.05).

To evaluate the antioxidant defense system in plants exposed to salt stress, the activities of four well-recognized kinds of antioxidant enzymes, SOD, CAT, POD and APX, were measured in leaves of both WT and transgenic plants. There were opposing trends for H_2_O_2_ and O_2_^•−^. As shown in **Figures 10A–D**, antioxidant enzyme activities between WT and transgenic plants without a salt treatment showed no significant differences. Nevertheless, a NaCl treatment increased the enzyme activities in all of the plants, with the enzyme activities being higher in ‘T_1_-71’ and ‘T_1_-68’ than in WT plants. During the experimental period, SOD, POD, CAT, and APX activities peaked at 8 days, were maintained at high levels, and finally decreased at day 16, but their levels remained high compared with the CK. When compared with WT plants after 8 days of treatment, the SOD activities in leaves of ‘T_1_-68’ and ‘T_1_-71’ plants increased by 7.1 and 12.3%, respectively (*P* < 0.01; **Figure 10A**), and the POD activities increased by 41.9 and 45.9%, respectively (*P* < 0.05; **Figure 10B**). The CAT activities increased by 26.2 and 27.1%, respectively (*P* < 0.05; **Figure 10C**), and the APX activities increased by 32.7 and 40.0% (*P* < 0.01; **Figure 10D**), respectively. Thus, *CsPLD*α-overexpression in tobacco plants facilitated the growth and salt-stress tolerance of plants.

### Soluble Sugar, Proline, and Soluble Protein Contents under Long-Term NaCl Stress

**FIGURE 11:**
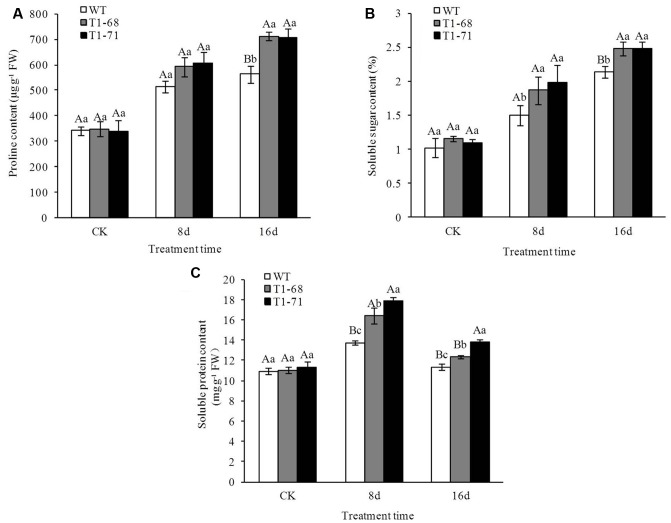
**Effects of long-term NaCl stress on the contents of proline** **(A)**, soluble sugar **(B)**, and soluble protein **(C)** in both WT and transgenic tobacco leaves. Values are means ± SD (*n* = 3). Mean values followed by different capital letters (A–C) and small letters (a–c) are significantly different (*P* < 0.01) and (*P* < 0.05), respectively.

The contents of soluble sugar, proline, and soluble protein in the leaves of the lines were determined (**Figure 11**), and their contents under salt stress were higher than in the CK. However, there were no significant differences between WT and transgenic plants at CK. The contents of proline and soluble sugar increased in a time-dependent manner and finally peaked at day 16, while the soluble protein content peaked at 8 days, was maintained at a high level, and finally decreased at day 16. After the 16-days treatment, the contents of proline and soluble sugar in leaves of ‘T_1_-68’ and ‘T_1_-71’ transgenic lines increased by 26.5 and 25.3% (**Figure 11A**), and 16.3 and 16.4% (**Figure 11B**), respectively, compared with WT. After the 8-days treatment, the content of soluble protein in leaves of ‘T_1_-68’ and ‘T_1_-71’ transgenic lines increased by 19.4% (*P* < 0.05) and 29.7% (**Figure 11C**), respectively, compared with WT. These results indicated that the substances capable of causing major osmotic adjustments were significantly upregulated by the overexpression of *CsPLD*α.

## Discussion

Under growth conditions, plants are frequently subjected to various stresses, especially high salinity, which severely affects crop production worldwide. PLDs may play crucial roles in plant responses to various environmental stresses (Bargmann et al., 2009). The functions of different PLD isoforms can be unique and overlapping, and may depend on their structural properties, regulatory functions and substrate specificities (Mao et al., 2007). In the present study, the *CsPLD*α-overexpression was positively correlated with the flourishing growth of tobacco plants in response to salt stress, because the expression of endogenous *NtPLDα1* had no difference between the WT and transgenic plants under short-term stress. Under a long-term 250 mM NaCl stress, the root FWs and DWs of transgenic plants increased (**Figure 5**), and membrane leakage decreased (**Figure 8**), compared with WT. It may be that Cs*PLD*α-overexpression improves the PLD activity directly (**Figure 6**) and that this kind of lipid hydrolyzing enzyme can hydrolyze membrane phospholipids to produce PA, which has both membrane degradation and signal transduction functions, acts as a secondary messenger and has evolved a signaling role that may mitigate stress injury (Itoh et al., 2009). The latter is the leading role compared with the membrane degradation function in some cases. In addition, our results were in accordance with those of previous studies (Gigon et al., 2004), demonstrating that membrane remodeling and rearrangements induced by drought stress aid plants in resisting drought injury, and PLD activity is involved in such membrane alterations. Under short-term stress (5 h), the PLD activities and PA contents in leaves of the transgenic lines significantly increased (**Figure 1**), and the level of *NtMAPK* expression in transgenic plants also increased. Thus, PA may act as a secondary messenger, playing a main role in binding to, and regulating the activities of, target proteins associated with salt-stress signaling (Anthony et al., 2004; Testerink et al., 2004; Yu et al., 2010; Galvan-Ampudia and Testerink, 2011; McLoughlin et al., 2013), especially in the *NtMAPK* signaling pathway. It could enhance the expression of transporter-related genes through Na^+^ or K^+^, as well as proton pump genes (**Figure 3**). Mitogen activated protein kinase 6 (MPK6) can be activated by PLDα1-derived PA directly under salt condition. Then, the Na^+^/H^+^ antiporter, salt overly sensitive 1 (SOS1), downstream of MPK6 is phosphorylated by this activated kinase directly. Thus, a lower level of MPK6 activity and higher Na^+^ accumulation are displayed in *PLDα1* mutants than in WT plants (Yu et al., 2010). Additionally, PLD-produced PA may also bind to, and regulate the activity of, other target proteins associated with salt-stress signaling, such as Snf-related protein kinases (SnRKs) (Testerink et al., 2004), 3-phosphoinositide-dependent kinase 1 (PDK1) (Anthony et al., 2004), and glyceraldehyde 3-phosphate dehydrogenase (GAPDH) (McLoughlin et al., 2013). The SnRKs gene family is activated by, and plays an important role in response to, osmotic stress (Hrabak et al., 2003; Boudsocq et al., 2004; Fujii et al., 2011). PDK1, another PA target, can be stimulated by *PLD*ζ*2*-produced PA, which in turn mediates the internalization of PIN2 to regulate salt avoidance (Galvan-Ampudia and Testerink, 2011). GAPDH is not only a classic glycolytic enzyme, but also a multifunctional protein involved in a number of cellular processes. GAPDH can selectively bind to inositol 1,4,5-trisphosphate receptors (IP3R), leading to the generation of NADH and regulation of intracellular Ca^2+^ signaling (Patterson et al., 2005). Moreover, PLD-derived PA may activate proton-pumps, like vacuolar H^+^-ATPase and H^+^-PPase, which confer a proton gradient for the Na^+^/H^+^ exchange to enhance salt tolerance (Zhang et al., 2006; Shen et al., 2011). A new study also suggested that there was a functional correlation between the activities of PLD and the H^+^-ATPase mediated by PA release (Muzi et al., 2016). In this study, we monitor the expression levels of genes related to ion exchange, including *NtNHX1*, *NtNKT1* and *NtHAK1*, and genes related to H^+^-ATPase, such as *NtNHA1* and *NtVAG1*, under short-term NaCl stress. The expression levels of all five genes in leaves and roots increased (**Figure 3**). Thus, maintaining Na^+^–K^+^ homeostasis helps to elevate salt tolerance.

Although the Na^+^–K^+^ homeostasis induced by PA plays an important role in assisting plant responses to salt stress, it is not the only way to assuage the damage to membrane systems caused by the high salinity stress. Salt stress can destroy the integrity of the cell membrane, resulting in the leakage of more solute (Yao et al., 2011). Another PLD-derived way to reduce salt stress damage is through the scavenging of ROS. Antioxidant enzymes, such as SOD, POD, CAT and APX, are employed to scavenge ROS to alleviate oxidative damage and enhance stress tolerance. However, although a high ROS concentration is harmful to plants, recent evidence suggests that ROS function as important physiological regulators of intracellular signaling pathways at certain concentrations (Apel and Hirt, 2004; Ray et al., 2012). It has been hypothesized that the involvement of PLD and PA in the production of, and response to, ROS may underlie the mechanisms by which PLD and PA mediate plant physiological processes. Thus, the SOD-synthesizing activity first increased and then decreased as PA concentrations increased (Sang et al., 2001; Zhang et al., 2005). In our study, the antioxidant enzyme activities of overexpression plants are much higher than those of the WT plants. In particular, the antioxidant enzyme activities of ‘T_1_-71’ are higher than those of ‘T_1_-68,’ which could be the result of different PA concentrations. Thus, ROS production, including H_2_O_2_ and O_2_^•−^, is much lower than in WT plant. The content of MDA, an important product of membrane lipid peroxidation, was lower than in the WT plant. The damage to the membrane system induced by salt stress was alleviated in overexpression plants compared with in WT plants. These defenses require a continuous consumption of energy, and this elevated energy production limits the further generation of ROS (Møller, 2001). Whether the enhanced ROS scavenging capacity is related to the improved ATP generation requires further investigation in PLD-overexpression plants.

In response to salt stress, many plants can accumulate compatible osmolytes, which facilitate osmotic adjustment leading to increasing dehydration tolerance during stress, such as proline (Liu et al., 2007) and soluble sugar (Boriboonkaset et al., 2013). The amino acid proline is a most important osmotic, which plays a major role in the stabilization of membranes and prevents degradation of proteins and enzymes under stress (Farooq et al., 2009). And soluble sugars are involved in osmoregulation mechanisms within the cell, controlling water potential and osmotic potential (Pattanagul and Thitisaksakul, 2008; Cha-um et al., 2009a,b). PLD also facilitates the generation of proline, soluble sugar, and soluble protein into vacuoles to maintain osmotic balance. The beneficial effect of proline hyperaccumulation on salt tolerance has been demonstrated in a range of halophyte species (Radyukina et al., 2007; Szabados and Savouré, 2010), and PLD functions as a positive regulator in *Thellungiella* (Ghars et al., 2012). Several studies indicated that the vacuolar Na^+^/H^+^ antiporter *AtNHX3* could enhance constituent soluble sugar contents under salt stress conditions in *Arabidopsis* (Liu et al., 2008). However, this enhanced solute accumulation balanced the solute potential, which then contributed to cell growth, and then increased both the FWs and DWs of overexpression plant roots. Solutes, like proline, may play other non-exclusive roles possibly to limit or repair damage due to stress (Hare et al., 1999; Maggio et al., 2002), and then elevate the tolerance to stress.

## Conclusion

**FIGURE 12:**
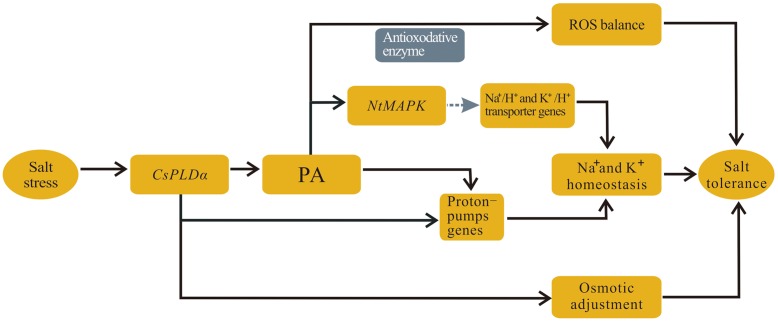
**Schematic illustration for main mechanism functioned by *CsPLD*α.** (1) *CsPLD*α and CsPLDα-produced PA can activate proton-pumps to maintain Na^+^–K^+^ homeostasis; (2) CsPLDα-derived PA also activate the scavenging of ROS to function in structural stabilization of membranes; (3) and *CsPLD*α facilitates the accumulation of osmoprotective compounds to maintain osmotic balance.

*CsPLD*α is expressed dominantly in vigorously growing tobacco cells under salt stress, both in leaves and roots, and its overexpression plant can improve the tolerance of tobacco plants to high salinity. Based on aforementioned results and analysis, a schematic illustration for a possible mechanism of *CsPLD*α in plant was produced (**Figure 12**). The main adaptive strategies to salt stress are: (1) *CsPLD*α and CsPLDα-produced PA can activate proton-pumps to maintain Na^+^–K^+^ homeostasis; (2) CsPLDα-derived PA also activate the scavenging of ROS to function in structural stabilization of membranes; (3) and *CsPLD*α facilitates the accumulation of osmoprotective compounds to maintain osmotic balance. In addition, we hypothesized that *NtMAPK* may be activated by CsPLDα-derived PA directly under salt conditions, and that *NtMAPK*, which is downstream of PA, can activate the Na^+^/H^+^ antiporter. This is beneficial to maintain Na^+^–K^+^ homeostasis and elevate salt tolerance. Further studies are needed to investigate whether other PLD-mediated lipid signaling cascades in plant growth are activated, and which functions play leading roles, as well as how the cell signaling in stress responses is regulated. Understanding these matters will aid in understanding the PLD-mediated lipid signaling cascades, development and stress responses.

## Author Contributions

Conceived and designed the research: FY, SL, and TJ. Performed the research: SL, TJ, MH, and QD. Analyzed the data: SL and TJ. Contributed regents/materials/analysis tools: XW, MW, QS, BG, and YL. Wrote the first draft of the manuscript: TJ. Improved the first draft of the manuscript: FY and SL. All authors have read and approved this manuscript.

## Conflict of Interest Statement

The authors declare that the research was conducted in the absence of any commercial or financial relationships that could be construed as a potential conflict of interest.

## Abbreviations

MAPK: mitogen activated protein kinase
PA: phosphatidic acid
PLD: phospholipase D
qRT-PCR: quantitative real-time polymerase chain reaction
ROS: reactive oxygen species
WT: wild type
