# Supplementary material for: Overexpression of Cucumber Phospholipase D alpha Gene (CsPLDα) in Tobacco Enhanced Salinity Stress Tolerance by Regulating Na+–K+ Balance and Lipid Peroxidation
Source: Front Plant Sci. 2017 Apr 7;8:499. doi: 10.3389/fpls.2017.00499 (PMC5383712; doi:10.3389/fpls.2017.00499)
Supplement: Supplementary file 1 [file Table_1.DOC]

**Supplementary material**

Table S1 Primers for real-time quantitative PCR

| Primer name | Primer sequence |
| --- | --- |
| *NtNHX1F* | 5'- CCGCTTGGCAATGGTATCC-3' |
| *NtNHX1R* | 5'- CGCTCCGTTCTGTTGGTG -3' |
| *NtNKT1F* | 5'- AATGCCTGGTGCTCCTTAC-3' |
| *NtNKT1R* | 5'- TTCTCCTCAATCTGCCGTTAG-3' |
| *NtHAK1F* | 5'- TGTATGGTTATTGGTGATGGAGTC-3' |
| *NtHAK1R* | 5'- ACACAAGCAACTGGAACTTCTA-3' |
| *NtNHA1F* | 5'- AGAAGGCAGACATTGGTATAGC-3' |
| *NtNHA1R* | 5'- AACACAGCACTCACGATAACA-3' |
| *NtVAG1F* | 5'- GCTTACATGGAGGCTGAGTT-3' |
| *NtVAG1R* | 5'- GGAGCATCTGGACAACATCA-3' |
| *NtMAPKF* | 5’- CCTATACCAAATATTACGAGGACTT -3’ |
| *NtMAPKR* | 5’- GATACCACCGCGTTACGAC -3’ |
| *NtPLDα1F* | 5’- CCATGTTTACGCATCACCAG-3’ |
| *NtPLDα1R* | 5’- CTCCCATCGCAAAGATCAAT-3’ |
| *β-actinF* | 5’- TTAAAGAGAAACTGGCATATGTTG-3’ |
| *β-actinR* | 5’- GCCCATCTGGTAA CTCATAGC-3’ |
